# The PSP subcortical atlas - a new neuroimaging atlas for progressive supranuclear palsy Richardson's syndrome

**DOI:** 10.1016/j.nicl.2026.104075

**Published:** 2026-09-29

**Authors:** Cassandra Marotta, Benjamin Sinclair, Meng Law, Terence J. O'Brien, Lucy Vivash

**Affiliations:** a Department of Neuroscience, School of Translational Medicine, Monash University, 99 Commercial Road, Melbourne, Victoria 3004, Australia; b Department of Radiology, Alfred Health, 55 Commercial Road, Melbourne, Victoria 3004, Australia; c Department of Electrical and Computer Systems Engineering, Monash University, Wellington Road, Clayton, Victoria 3800, Australia; d Department of Neurology, Alfred Health, 55 Commercial Road, Melbourne, Victoria 3004, Australia

**Keywords:** Progressive supranuclear palsy, Neuroimaging atlas, Basal ganglia, Subcortical segmentation

## Abstract

**Objectives:**

The severe cerebral atrophy patterns of progressive supranuclear palsy (PSP) create challenges in clinical neuroradiology practice and neuroimaging research as often, subcortical regions are not accurately segmented with existing atlases or require labour intense manual segmentation. We aimed to create a PSP specific neuroimaging atlas.

**Methods:**

Seven subcortical regions were manually segmented on Quantitative Susceptibility Mapping (QSM) images of 33 PSP Richardson's Syndrome (PSP-RS) subjects and used to automatically segment 10 validation subjects using the ANTs Joint Label Fusion module. Accuracy was confirmed by determining the Dice coefficient (DC) and 95% Hausdorff Distance (HD95) using manual segmentation of QSM images of as the gold standard in a leave-one-out cross validation (LOOCV).

**Results:**

The PSP atlas outperformed existing atlases with mean DC and HD95 of 0.90 ± 0.20 and 0.86 mm ± 0.17 compared to 0.73 ± 0.04 and 2.69 mm ± 0.75 for Talaraich and Harvard-Oxford atlases.

**Conclusions:**

Our new PSP subcortical atlas outperforms currently available atlases and can be applied to multimodal datasets and T1-weighted images alone.

## Highlights

• Severe atrophy in PSP leads to poor coregistration of many neuroimaging atlases.
• The PSP atlas was developed on manual segmentation of 43 PSP-RS subjects.
• The PSP atlas significantly outperformed existing atlases and segmentation tools.
• The PSP atlas can be applied on multimodal data and T1-weighted images alone.

## 1 Introduction

Progressive supranuclear palsy (PSP) is a rare, neurodegenerative movement disorder, affecting approximately 6 per 100,000 people (O'Sullivan et al., 2008; Respondek et al., 2014; Schrag et al., 1999; Williams et al., 2005). It is a rapidly progressing, inevitably fatal disease, with the most common phenotype, PSP Richardson's syndrome (PSP-RS) having a survival rate of approximately 6–7 years from symptom onset (O'Sullivan et al., 2008; Respondek et al., 2014; Schrag et al., 1999; Williams et al., 2005).

PSP is associated with the accumulation of hyperphosphorylated tau in specific brain regions, leading to severe tau aggregation and regional brain atrophy. In PSP-RS this typically begins in subcortical areas of the basal ganglia before propagating to areas of the cerebellum, parietal and frontal lobes (Williams and Lees, 2009). There are currently no cures, treatments or objective biomarkers available that are specific for PSP (Coughlin and Litvan, 2022). Considerable research is being done to identify neuroimaging biomarkers, including the use of tau PET, structural MRI and advanced MRI techniques such as quantitative susceptibility mapping (QSM) (Marotta et al., 2025). However, identifying early biomarkers of disease is challenging, in part due to a delay of approximately 3–4 years between the initial symptom onset and diagnosis (Respondek et al., 2013). During this time significant neurodegeneration and clinical decline has already occurred, such that atrophy or hypometabolism of the midbrain can already be observed at time of diagnosis, leading to an increased diagnostic confidence labelled “imaging supported diagnosis” (Hoglinger et al., 2017). In many research studies, the mean disease duration is often greater than three years and disease severity scores on the PSP rating scale (PSPRS) are above that considered “mild disease” (score < 30) (Golbe and Ohman-Strickland, 2007; Marotta et al., 2025). In studies investigating brain volume many brain regions exhibit noticeable atrophy compared to healthy controls at baseline assessment (Höglinger et al., 2017).

The severe atrophy patterns of PSP create challenges in clinical neuroradiology practice and neuroimaging research as existing brain atlases, typically developed using data from healthy subjects, co-register poorly to PSP subjects, particularly in subcortical regions and areas close to the ventricles which are most susceptible to disease-related atrophy.

Atlas based volumetry (ABV) studies often investigate regional brain atrophy in PSP and is recommended for use as outcome measures in clinical trials (Höglinger et al., 2017). PSP studies that look at regional atrophy rates often use the Harvard-Oxford subcortical atlas (Desikan et al., 2006; Frazier et al., 2005; Goldstein et al., 2007; Lancaster et al., 2000, Lancaster et al., 2007; Makris et al., 2006) for regions such as the caudate, putamen and thalamus (Höglinger et al., 2017; Huppertz et al., 2016; Talai et al., 2019, Talai et al., 2021). In part due to difficulties in accurate delineations, regions such as the globus pallidus, red nucleus, substantia nigra and subthalamic nucleus, which are key nuclei associated with tau accumulation and atrophy in PSP are not commonly included in ABV studies. The few recent exceptions have used a specific basal ganglia atlas however this atlas was also developed using a small cohort of healthy participants and is therefore susceptible to the same co-registration issues as other healthy brain atlases when applied to PSP cohorts (Keuken et al., 2017; Quattrone et al., 2024, Quattrone et al., 2025).

QSM images provide the distinct advantage over other MRI sequences in that they have increased contrast within the subcortical nuclei, enabling the visualisation and accurate delineation of these regions. However, existing PSP studies investigating QSM images generally use manual segmentations rather than brain atlases (Haji et al., 2025; Krishnan et al., 2023; Mazzucchi et al., 2019, Mazzucchi et al., 2022; Zhang et al., 2022). Manual segmentations are labour intense and time consuming, meaning they cannot feasibly be applied to large datasets such as those required in clinical trials. Despite this, it is uncommon for studies investigating QSM in PSP to use a brain atlas and when atlases have been used, multiple have been required for identification and delineation of all regions of interest (Satoh et al., 2023, Satoh et al., 2025; Weih et al., 2025). This highlights the limitations of currently available atlases and the implications of suboptimal accuracy and reliability in PSP studies, cautioning against their use for ABV and other studies where these regions cannot be accurately visualised for quality assessment. Furthermore, the acquisition sequences required for QSM are not routinely included in standard clinical and research MRI protocols and therefore manual segmentation of these regions is not always possible.

Due to the current challenges and limitations associated with both automated and manual segmentation of subcortical regions this work aimed to create a PSP-specific subcortical atlas of the basal ganglia and associated regions that can be used for accurate regional analysis of neuroimaging data, irrespective of QSM availability.

## 2 Methods

### 2.1 Cohort

We included a total of 43 subjects with a diagnosis of probable PSP-RS according to the MDS criteria (Hoglinger et al., 2017). The mean age was 67.8 years (SD 6.8, range 47–79) and 17 subjects were female. The mean disease duration was 1.8 (SD 2.0, range 0–6) years since diagnosis and mean PSP rating scale (PSPRS) score was 31.2 (SD 10.4, range 8–56). Subjects were recruited as part of a clinical trial (Alfred Health HREC number 594/20, ACTRN12620001254987)(Vivash et al., 2021) and written informed consent was obtained from all participants for being included in the study. All subjects underwent MRI scanning at six sites across Australia.

### 2.2 MRI acquisitions

MRI scans were acquired on a 3 T Siemens or GE scanner. 3D T1-weighted magnetization-prepared rapid acquisition gradient echo (MPRAGE) (sagittal plane; 0.8 mm voxel resolution; 320 slices; 0.8 mm slice thickness) were acquired for all subjects. 3D T2*-weighted multi-echo gradient echo sequences or 3D-enhanced fast gradient echo Susceptibility Weighted Angiography (SWAN) sequences were acquired (axial plane; 0.8 mm isotropic voxel resolution; 0.8 mm slice thickness). Full acquisitions parameters for each site are detailed in Supplementary Table 1.

### 2.3 Image processing

T2* and SWAN data was processed to reconstruct the QSM images using the SEPIA toolbox (version 1.2.2.6)(Chan and Marques, 2021) in Matlab (R2024b). Brain masks of the magnitude images were obtained using FMRIB's brain extraction tool (BET)(Smith, 2002). The multi-echo phase data were combined using optimal-weighting and Laplacian phase unwrapping was completed using the STI suite algorithm (v3.0)(Li et al., 2011; Schweser et al., 2017). Background field removal was performed with the Variable-kernel Sophisticated Harmonic Artefact Reduction for Phase data (VSHARP) algorithm from the STI suite with a 12 mm spherical mean value (SMV) kernel size (Li et al., 2011). Quantitative susceptibility maps were then reconstructed with QSMnet+ using the CSF as a reference tissue (Jung et al., 2020, Jung et al., 2022; Yoon et al., 2018). QSM images were then non-linearly registered to each subjects native T1-weighted image using Advanced Normalization Tools (ANTs)(version 2.4.3) (Avants et al., 2008).

### 2.4 Atlas development

QSM images were opened in FSLeyes and thresholded at −0.2–0.2 ppm. FSLeyes was used to manually segment the caudate, putamen, globus pallidus, substantia nigra, subthalamic nucleus, red nucleus, and thalamus on 33 subjects. The manual segmentations were reviewed by a senior neuroradiologist (ML). The manual segmentations of all regions of interest (ROIs) were then collated to create a ROI label image for each subject.

A PSP-specific template was created by iteratively aligning a sub-cohort of 20 subjects T1w images with symmetric diffeomorphic registration within the ANTs multivariate template construction script (Avants et al., 2008).

Each labelled subject's T1w image was then co-registered to the PSP-template space using ANTs (version 2.4.3) with rigid, affine, and nonlinear (SyN) transformations and mutual information as the similarity metric. The QSM and ROI label images were then warped to the PSP-template using the resulting affine and nonlinear transformations with nearest-neighbour interpolation to preserve label integrity. The co-registered T1w, QSM and ROI label images were subsequently used as labelled inputs in the ANTs Joint Label Fusion (JLF) module to automatically segment ten unlabelled validation subjects (Avants et al., 2008; Wang and Yushkevich, 2013). The ANTs JLF algorithm registered each unlabelled subject to every labelled subject's T1w and QSM images. The corresponding ROI labels from the labelled subjects were then combined using a similarity-weighted voting scheme, giving greater influence to the subjects whose images most closely match the unlabelled subject, creating the ROI segmentation for the unlabelled subject. The unlabelled subjects' segmentation images were then transformed back into T1w native space.

### 2.5 Atlas validation

The PSP atlas was compared against commonly used healthy brain atlas' (a combination of the Talaraich atlas [substantia nigra, globus pallidus, subthalamic nucleus and red nucleus] and Harvard-Oxford subcortical atlas [putamen, caudate and thalamus])(Desikan et al., 2006; Frazier et al., 2005; Goldstein et al., 2007; Lancaster et al., 2000, Lancaster et al., 2007; Makris et al., 2006). These ROIs were obtained in FSLeyes in MNI space, non-linearly co-registered to the PSP-template space using FMRIB's non-linear image registration tool and then the inverse warp from the each subjects' T1w co-registration to PSP-template was applied to the ROIs with nearest neighbour interpolation to obtain the native ROIs (Andersson et al., 2026; Jenkinson et al., 2012). FreeSurfer's (version 7.4.0) automated segmentation (aseg) was also included as it is a common segmentation method, however this only includes ROIs of the putamen, caudate, globus pallidus and thalamus. FreeSurfer's recon-all was performed and the resulting aseg files were used to obtain the binarized ROIs. The ROIs were then resampled to each subjects' native T1w image using FreeSurfer's mri_vol2vol with nearest-neighbour interpolation (Fischl, 2012; Fischl et al., 2002).

Three versions of the PSP atlas segmentations were assessed, T1w only (both labelled and unlabelled subjects have T1w inputs only), Hybrid (T1w + QSM labelled inputs, T1w only unlabelled inputs) and multimodal (both labelled and unlabelled subjects have T1w and QSM inputs).

Accuracy of the automatic PSP atlas segmentations was confirmed in the ten validation subjects by manual segmentation of the QSM images as the gold standard (Fig. 1). The three versions of the segmentations and the best performing current atlas (a combination of the Talaraich atlas [substantia nigra, globus pallidus, subthalamic nucleus and red nucleus] and Harvard-Oxford subcortical atlas [putamen, caudate and thalamus])(Desikan et al., 2006; Frazier et al., 2005; Goldstein et al., 2007; Lancaster et al., 2000, Lancaster et al., 2007; Makris et al., 2006) were compared to the gold standard manual segmentations. Dice coefficient (DC) and 95% Hausdorff Distance (HD95) were computed in python (version 3.8) for each ROI per atlas and compared to the gold standard manual segmentations. A combined “All ROIs” metric was also computed by merging all ROI masks available for each atlas (the STN, SN and RN are not segmented in FreeSurfer). To mitigate the small sample size and statistical power of the validation cohort we then further assessed atlas performance using a leave-one-out cross validation (LOOCV) approach on the full 43 subjects, where each subject was treated as an unlabelled subject and segmented once using the remaining 42 subjects as labelled inputs.

**Fig. 1:**
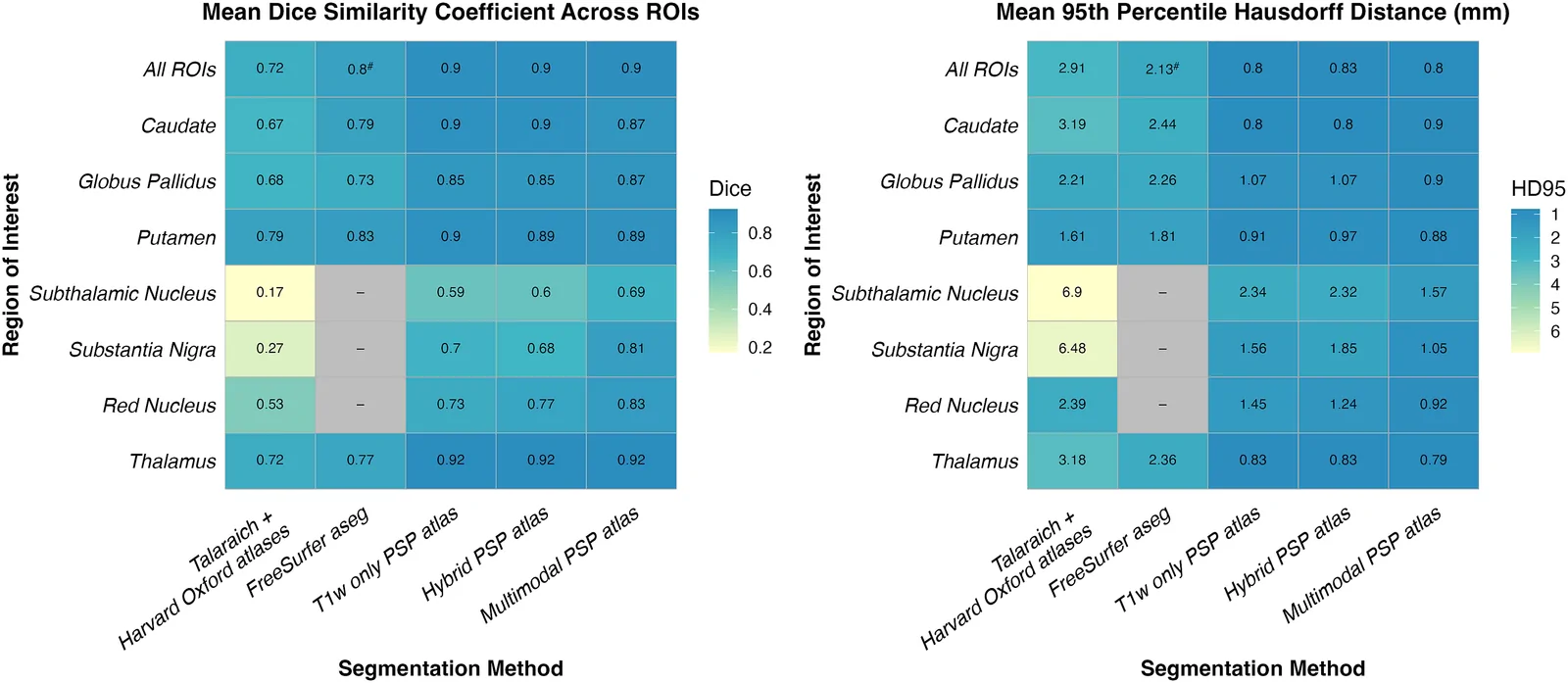
Comparison of PSP-specific atlas performance to gold-standard manual segmentations. *# Mean based on all available regions (caudate, globus pallidus, putamen, thalamus).*

### 2.6 Statistical analyses

Statistical analyses were conducted using Rstudio (version 2024.09.0 + 375). Descriptive statistics were computed for the DC and HD95 of each atlas type. To compare accuracy across the atlas types one-way repeated-measures ANOVAs were conducted for each ROI × metric combination. Pairwise post-hoc comparisons were then performed using Tukey's Honest Significant Difference (HSD) test, controlling for the familywise error rate across all pairwise atlas comparisons. Data are presented as mean ± standard deviation unless stated otherwise.

### 2.7 Data availability statement

The manual segmentations and code for the T1w only and multimodal versions of the PSP atlas are being prepared as an Apptainer container from https://github.com/iBrain-Lab/PSP_atlas.

## 3 Results

There were 33 subjects included in the labelled segmentation cohort with a mean PSPRS score of 32.1 (SD 10.1, range 8–56) and disease duration of 1.9 (SD 2.2, range 0–6) years. The validation cohort consisted of ten subjects from six sites with a mean PSPRS score of 28.1 (SD 11.6, range 11–44) and disease duration of 1.2 (SD 1.55, range 0–4) years.

The descriptive statistics for each atlases individual ROI and combined ROIs DC and HD95 compared to the manual segmentations are presented in Table 1. Mean DC and HD95 scores were comparable across imaging sites (data not shown). A limitation of the HD95 scores is the floor effect equal to the resolution of the voxel size. Therefore, many of the HD95 scores for the PSP atlas ROIs are reported at 0.8 mm because any differences smaller than that cannot be delineated.

**Table 1:** Descriptive statistics of the PSP atlas, Talaraich+Harvard-Oxford atlas and FreeSurfer aseg compared to gold standard manual segmentations.

Atlas	ROI	metric	n	mean	SD	CI lower	CI upper	median	IQR	min	max
Multimodal PSP atlas	Caudate	DC	43	0.850	0.053	0.834	0.867	0.867	0.035	0.621	0.920
		HD95	43	1.051	0.426	0.920	1.182	0.800	0.331	0.800	2.530
	Globus Pallidus	DC	43	0.867	0.036	0.856	0.878	0.871	0.038	0.771	0.923
		HD95	43	0.963	0.373	0.848	1.077	0.800	0.000	0.800	2.400
	Putamen	DC	43	0.887	0.023	0.880	0.894	0.889	0.025	0.828	0.925
		HD95	43	0.889	0.209	0.825	0.954	0.800	0.000	0.800	1.600
	Red nucleus	DC	43	0.827	0.059	0.809	0.845	0.839	0.043	0.604	0.925
		HD95	43	0.958	0.361	0.847	1.069	0.800	0.331	0.800	2.884
	Substantia nigra	DC	43	0.792	0.062	0.773	0.811	0.806	0.069	0.611	0.910
		HD95	43	1.183	0.523	1.022	1.344	1.131	0.331	0.800	3.200
	Subthalamic nucleus	DC	43	0.711	0.067	0.691	0.732	0.714	0.092	0.548	0.822
		HD95	43	1.653	0.623	1.461	1.845	1.600	0.828	0.800	4.079
	Thalamus	DC	43	0.914	0.023	0.907	0.921	0.916	0.024	0.839	0.956
		HD95	43	0.841	0.193	0.782	0.901	0.800	0.000	0.000	1.386
	Combined ROIs	DC	43	0.896	0.020	0.890	0.902	0.901	0.022	0.838	0.930
		HD95	43	0.863	0.173	0.810	0.916	0.800	0.000	0.800	1.600
T1w only PSP atlas	Caudate	DC	43	0.883	0.046	0.868	0.897	0.894	0.019	0.706	0.919
		HD95	43	1.010	0.623	0.818	1.202	0.800	0.000	0.800	3.299
	Globus Pallidus	DC	43	0.831	0.037	0.819	0.842	0.838	0.044	0.745	0.899
		HD95	43	1.210	0.402	1.086	1.334	1.131	0.000	0.800	2.530
	Putamen	DC	43	0.894	0.022	0.888	0.901	0.901	0.026	0.826	0.928
		HD95	43	0.894	0.223	0.826	0.963	0.800	0.000	0.800	1.600
	Red nucleus	DC	43	0.758	0.066	0.738	0.778	0.769	0.070	0.500	0.852
		HD95	43	1.271	0.457	1.131	1.412	1.131	0.501	0.800	3.200
	Substantia nigra	DC	43	0.705	0.065	0.685	0.725	0.714	0.071	0.535	0.822
		HD95	43	1.631	0.517	1.472	1.790	1.600	0.658	0.800	3.200
	Subthalamic nucleus	DC	43	0.627	0.069	0.606	0.648	0.635	0.090	0.432	0.767
		HD95	43	2.085	0.631	1.890	2.279	1.960	0.474	1.131	3.752
	Thalamus	DC	43	0.918	0.016	0.913	0.922	0.922	0.016	0.873	0.938
		HD95	43	0.831	0.097	0.801	0.861	0.800	0.000	0.800	1.131
	Combined ROIs	DC	43	0.897	0.016	0.892	0.902	0.901	0.017	0.837	0.917
		HD95	43	0.876	0.231	0.805	0.947	0.800	0.000	0.800	1.789
Talaraich + Harvard-Oxford	Caudate	DC	43	0.682	0.069	0.660	0.703	0.691	0.111	0.512	0.810
		HD95	43	3.145	1.319	2.739	3.551	2.530	1.931	1.131	6.450
	Globus Pallidus	DC	43	0.674	0.038	0.662	0.686	0.676	0.058	0.579	0.741
		HD95	43	2.234	0.318	2.136	2.332	2.263	0.440	1.600	2.993
	Putamen	DC	43	0.802	0.041	0.790	0.815	0.809	0.052	0.675	0.859
		HD95	43	1.497	0.397	1.375	1.620	1.600	0.658	0.800	2.653
	Red nucleus	DC	43	0.521	0.068	0.500	0.542	0.526	0.092	0.306	0.628
		HD95	43	2.481	0.514	2.323	2.639	2.433	0.450	1.789	4.079
	Substantia nigra	DC	43	0.269	0.044	0.255	0.282	0.263	0.040	0.190	0.399
		HD95	43	5.981	0.921	5.698	6.264	6.043	1.255	3.299	7.632
	Subthalamic nucleus	DC	43	0.177	0.079	0.153	0.201	0.178	0.117	0.028	0.327
		HD95	43	6.334	1.269	5.944	6.725	6.299	1.601	4.079	9.925
	Thalamus	DC	43	0.742	0.040	0.729	0.754	0.737	0.036	0.671	0.846
		HD95	43	2.899	0.632	2.705	3.094	2.993	0.834	1.600	4.157
	Combined ROIs	DC	43	0.733	0.037	0.721	0.744	0.734	0.052	0.633	0.801
		HD95	43	2.689	0.753	2.457	2.921	2.530	0.882	1.600	4.800
FreeSurfer	Caudate	DC	43	0.805	0.041	0.792	0.817	0.814	0.060	0.701	0.862
		HD95	43	2.210	1.373	1.788	2.633	1.600	1.576	0.800	7.151
	Globus Pallidus	DC	43	0.692	0.074	0.669	0.715	0.703	0.113	0.445	0.815
		HD95	43	3.476	6.116	1.594	5.358	2.530	1.150	1.386	42.365
	Putamen	DC	43	0.819	0.036	0.808	0.830	0.825	0.043	0.689	0.869
		HD95	43	1.838	0.494	1.686	1.990	1.789	0.663	0.800	3.200
	Thalamus	DC	43	0.770	0.023	0.764	0.777	0.769	0.030	0.705	0.822
		HD95	43	2.365	0.258	2.285	2.444	2.400	0.267	1.789	2.884
	Combined ROIs	DC	43	0.804	0.010	0.801	0.807	0.805	0.014	0.782	0.832
		HD95	43	2.104	0.320	2.005	2.202	1.960	0.388	1.600	3.299

95% Hausdorff Distance (HD95), Dice Coefficient (DC); HD95 values are reported in mm.

### 3.1 PSP atlas versions

All three PSP atlas versions were assessed in the validation cohort of ten subjects. There were minimal differences between the three PSP atlas versions. The mean DC and HD95 scores of the multimodal, T1w only and hybrid versions were 0.90 ± 0.02 and 0.8 mm ± 0.0; 0.9 ± 0.01 and 0.8  mm ± 0.0; and 0.90 ± 0.01 and 0.83 mm ± 0.10, respectively (Fig. 2). In the validation cohort, post hoc Tukey's HSD comparisons revealed that the red nucleus was the only ROI showing a significant difference between the Hybrid and T1w only versions, with T1w only performing better on both DC (Δ = −0.040, SE = 0.011, t(27) = −3.49, *p* = .009) and HD95 (Δ = 1.46 mm, SE mm = 0.12, t(27) = 12.21, *p* < .0001). We therefore did not include the Hybrid version in subsequent LOOCV analyses.

**Fig. 2:**
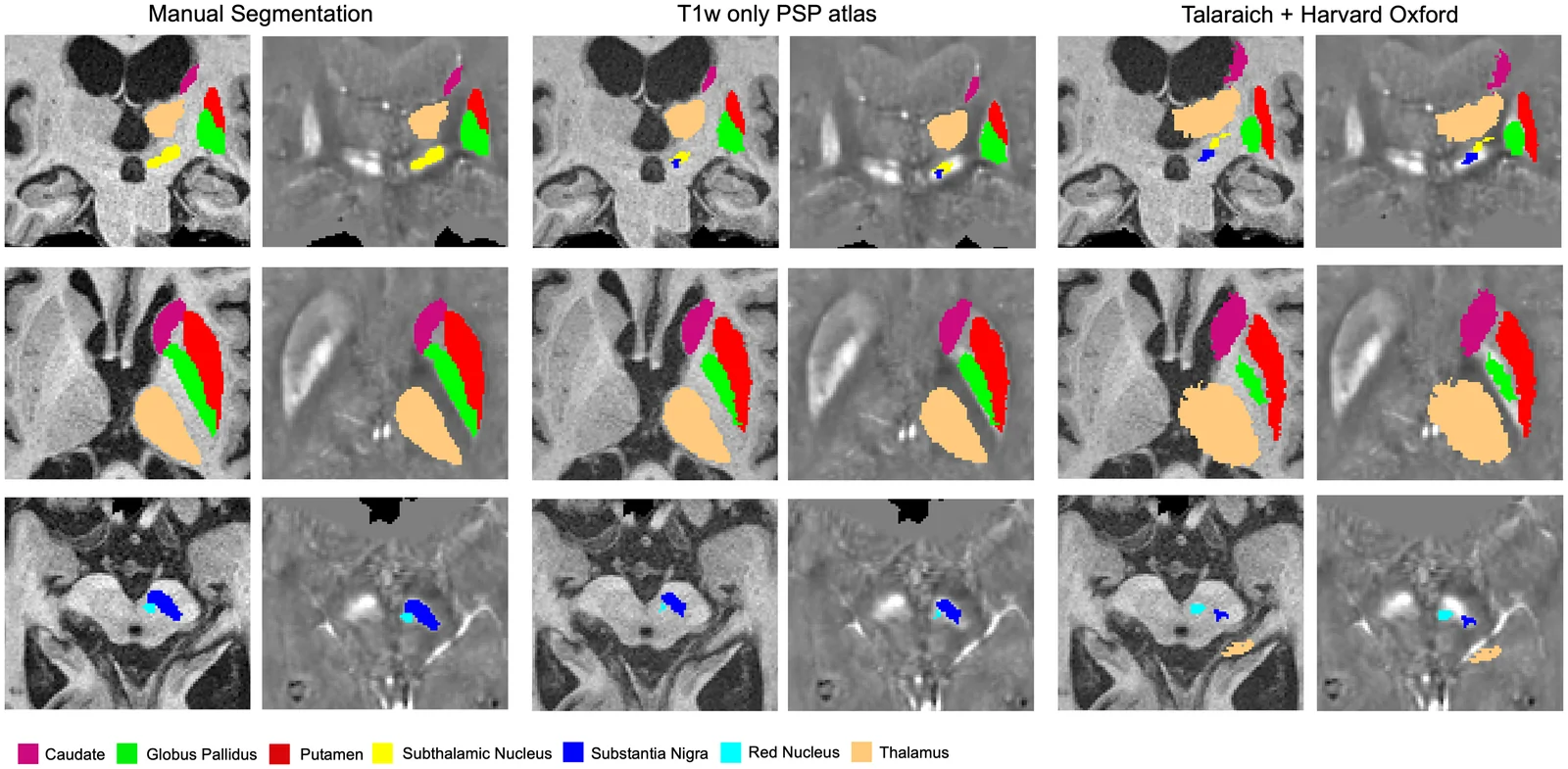
Visual comparison of T1w only PSP-specific atlas to manual segmentations and Talaraich + Harvard-Oxford atlases. *Coronal (top row) and axial (bottom two rows) comparison of subcortical regions of interest from manual segmentation, T1w only PSP atlas and Talaraich* *+* *Harvard-Oxford atlas* overlaid on T1w and QSM images.

In the LOOCV post hoc Tukey's HSD analyses, the Multimodal version exhibited small but significant improvements compared to the T1w only version in the DC for the globus pallidus (Δ = 0.036, SE = 0.009, t(126) = 4.04, *p* = .0005); DC and HD95 for the red nucleus (Δ = 0.069, SE = 0.008, t(84) = 8.52, *p* < .0001 and Δ = −0.314 mm, SE = 0.064 mm, t(84) = 8.52, *p* < .0001, respectively); DC and HD95 for the substantia nigra (Δ = 0.087, SE = 0.010, t(84) = 8.62, *p* < .0001 and Δ = −0.448 mm, SE = 0.150 mm, t(84) = −2.99, *p* = .010, respectively); and the DC for the subthalamic nucleus (Δ = 0.008, SE = 0.014, t(84) = 5.95, *p* < .0001). The T1w only version performed significantly better than the Multimodal version in the DC for the caudate (Δ = 0.032, SE = 0.010, t(126) = 3.090, *p* = .013). There were no significant differences between the Multimodal and T1w only versions in the putamen or thalamus (Table 2).

**Table 2:** Significant differences between the PSP atlas, Talaraich+Harvard-Oxford atlas and FreeSurfer aseg.

ROI	Comparison	metric	Δ	SE	df	t ratio	Adj pval
Combined ROIs	Freesurfer - Talaraich+Harvard-Oxford	DC	0.072	0.005	126	14.576	<0.001***
		HD95	−0.585	0.092	126	−6.351	<0.001***
	Multimodal PSP - Freesurfer	DC	0.092	0.005	126	18.656	<0.001***
		HD95	−1.240	0.092	126	−13.460	<0.001***
	Multimodal PSP - Talaraich+Harvard-Oxford	DC	0.163	0.005	126	33.233	<0.001***
		HD95	−1.826	0.092	126	−19.811	<0.001***
	Multimodal - T1w only PSP	DC	−0.001	0.005	126	−0.176	0.998
		HD95	−0.013	0.092	126	−0.136	0.999
	T1w only PSP - Freesurfer	DC	0.093	0.005	126	18.832	<0.001***
		HD95	−1.228	0.092	126	−13.323	<0.001***
	T1w only PSP - Talaraich+Harvard-Oxford	DC	0.164	0.005	126	33.409	<0.001***
		HD95	−1.813	0.092	126	−19.675	<0.001***
Caudate	Freesurfer - Talaraich+Harvard-Oxford	DC	0.123	0.010	126	11.769	<0.001***
		HD95	−0.934	0.203	126	−4.612	<0.001***
	Multimodal PSP - Freesurfer	DC	0.046	0.010	126	4.361	<0.001***
		HD95	−1.159	0.203	126	−5.722	<0.001***
	Multimodal PSP - Talaraich+Harvard-Oxford	DC	0.169	0.010	126	16.130	<0.001***
		HD95	−2.094	0.203	126	−10.333	<0.001***
	Multimodal - T1w only PSP	DC	−0.032	0.010	126	−3.090	0.013*
		HD95	0.041	0.203	126	0.204	0.997
	T1w only PSP - Freesurfer	DC	0.078	0.010	126	7.451	<0.001***
		HD95	−1.201	0.203	126	−5.926	<0.001***
	T1w only PSP - Talaraich+Harvard-Oxford	DC	0.201	0.010	126	19.220	<0.001***
		HD95	−2.135	0.203	126	−10.537	<0.001***
Globus pallidus	Freesurfer - Talaraich+Harvard-Oxford	DC	0.018	0.009	126	1.993	0.196
		HD95	1.242	0.654	126	1.899	0.234
	Multimodal PSP - Freesurfer	DC	0.175	0.009	126	19.577	<0.001***
		HD95	−2.514	0.654	126	−3.843	0.001**
	Multimodal PSP - Talaraich+Harvard-Oxford	DC	0.193	0.009	126	21.570	<0.001***
		HD95	−1.272	0.654	126	−1.944	0.215
	Multimodal - T1w only PSP	DC	0.036	0.009	126	4.036	0.001***
		HD95	−0.248	0.654	126	−0.378	0.981
	T1w only PSP - Freesurfer	DC	0.139	0.009	126	15.541	<0.001***
		HD95	−2.266	0.654	126	−3.464	0.004**
	T1w only PSP - Talaraich+Harvard-Oxford	DC	0.157	0.009	126	17.533	<0.001***
		HD95	−1.024	0.654	126	−1.565	0.402
Putamen	Freesurfer - Talaraich+Harvard-Oxford	DC	0.016	0.006	126	2.772	0.032*
		HD95	0.340	0.062	126	5.511	<0.001***
	Multimodal PSP - Freesurfer	DC	0.069	0.006	126	11.703	<0.001***
		HD95	−0.949	0.062	126	−15.352	<0.001***
	Multimodal PSP - Talaraich+Harvard-Oxford	DC	0.085	0.006	126	14.475	<0.001***
		HD95	−0.608	0.062	126	−9.841	<0.001***
	Multimodal - T1w only PSP	DC	−0.007	0.006	126	−1.196	0.631
		HD95	−0.005	0.062	126	−0.081	1.000
	T1w only PSP - Freesurfer	DC	0.076	0.006	126	12.899	<0.001***
		HD95	−0.944	0.062	126	−15.271	<0.001***
	T1w only PSP - Talaraich+Harvard-Oxford	DC	0.092	0.006	126	15.670	<0.001***
		HD95	−0.603	0.062	126	−9.760	<0.001***
Thalamus	Freesurfer - Talaraich+Harvard-Oxford	DC	0.029	0.006	126	4.803	<0.001***
		HD95	−0.535	0.079	126	−6.771	<0.001***
	Multimodal PSP - Freesurfer	DC	0.144	0.006	126	24.035	<0.001***
		HD95	−1.523	0.079	126	−19.293	<0.001***
	Multimodal PSP - Talaraich+Harvard-Oxford	DC	0.172	0.006	126	28.838	<0.001***
		HD95	−2.058	0.079	126	−26.063	<0.001***
	Multimodal - T1w only PSP	DC	−0.004	0.006	126	−0.596	0.933
		HD95	0.010	0.079	126	0.132	0.999
	T1w only PSP - Freesurfer	DC	0.147	0.006	126	24.630	<0.001***
		HD95	−1.534	0.079	126	−19.425	<0.001***
	T1w only PSP - Talaraich+Harvard-Oxford	DC	0.176	0.006	126	29.434	<0.001***
		HD95	−2.068	0.079	126	−26.195	<0.001***
Red nucleus	Multimodal PSP - Talaraich+Harvard-Oxford	DC	0.306	0.008	84	37.591	<0.001***
		HD95	−1.523	0.064	84	−23.859	<0.001***
	Multimodal - T1w only PSP	DC	0.069	0.008	84	8.519	<0.001***
		HD95	−0.314	0.064	84	−4.913	<0.001***
	T1w only PSP - Talaraich+Harvard-Oxford	DC	0.237	0.008	84	29.072	<0.001***
		HD95	−1.209	0.064	84	−18.946	<0.001***
Substantia nigra	Multimodal PSP - Talaraich+Harvard-Oxford	DC	0.523	0.010	84	51.830	<0.001***
		HD95	−4.798	0.150	84	−32.026	<0.001***
	Multimodal - T1w only PSP	DC	0.087	0.010	84	8.622	<0.001***
		HD95	−0.448	0.150	84	−2.991	0.010*
	T1w only PSP - Talaraich+Harvard-Oxford	DC	0.436	0.010	84	43.209	<0.001***
		HD95	−4.350	0.150	84	−29.036	<0.001***
Subthalamic nucleus	Multimodal PSP - Talaraich+Harvard-Oxford	DC	0.535	0.014	84	37.636	<0.001***
		HD95	−4.681	0.184	84	−25.446	<0.001***
	Multimodal - T1w only PSP	DC	0.084	0.014	84	5.945	<0.001***
		HD95	−0.432	0.184	84	−2.347	0.055
	T1w only PSP - Talaraich+Harvard-Oxford	DC	0.450	0.014	84	31.691	<0.001***
		HD95	−4.250	0.184	84	−23.100	<0.001***

Results from the ANOVA's post hoc Tukey's HSD analyses. 95% Hausdorff Distance (HD95), Dice Coefficient (DC); HD95 values are reported in mm. **4.**

### 3.2 PSP atlas versus existing ‘healthy brain’ atlases

The post hoc Tukey's HSD analyses revealed that both the Multimodal and T1w only versions of the PSP atlas significantly outperformed FreeSurfer and the Talaraich+Harvard-Oxford atlases (Table 2). Based on the combined ROIs the T1w only PSP atlas exhibited significant improvements in DC and HD95 compared to FreeSurfer's aseg (Δ = 0.093, SE = 0.005, t(126) = 18.83, *p* < .0001 and Δ = −1.228 mm, SE = 0.092 mm, t(126) = −13.32, *p* < .0001) and the Talariach+Harvard-Oxford atlas (Δ = 0.16, SE = 0.005, t(126) = 33.41, *p* < .0001 and Δ = −1.81 mm, SE = 0.092 mm, t(126) = −19.68, *p* < .0001).

### 3.3 Individual ROI performance

The most accurately segmented ROI was the thalamus with DC and HD95 scores of 0.92 ± 0.02 and 0.83 mm ± 0.1 in the T1w only PSP atlas and a DC score of 0.91 ± 0.02 in the Multimodal atlas. The PSP atlas segmentation performed worst on the subthalamic nucleus where the best DC and HD95 scores were 0.71 ± 0.07 and 1.65 mm ± 0.62 for the Multimodal version. However, this was still largely better than the Talaraich+Harvard-Oxford atlas in which the DC and HD95 were 0.18 ± 0.08 and 6.33 mm ± 1.27. Four of the seven ROIs obtained DCs above 0.8 in the T1w only atlas, this increased to five ROIs in the multimodal PSP atlas while the Talaraich+Harvard-Oxford atlas and FreeSurfer aseg only achieved DC above 0.8 in the putamen and caudate (Table 1).

## 4 Discussion

We curated a collection of gold standard segmentations of subcortical regions in a cohort of PSP-RS subjects that can be used to segment unlabelled MRI data, creating a PSP subcortical atlas.

Three versions of the PSP atlas were assessed and the multimodal version was the best performing. This option should be utilized when QSM images of the unlabelled subjects are available. However, the MRI acquisition sequences required for QSM such as T2* or SWAN sequences are not commonly included in standard MRI protocols and require advanced image processing pipelines. Therefore, a major advantage of the PSP-subcortical atlas is the T1w only version. While T1-weighted images are routinely acquired in MRI protocols many subcortical regions such as the red nucleus and substantia nigra cannot be clearly delineated on these images and therefore cannot be manually segmented. The T1w only version of the PSP subcortical atlas allows for these regions to be studied with basic structural MRI protocols that are largely available and do not require advanced acquisition sequences or image processing. The T1w only version of the PSP atlas significantly outperformed FreeSurfer and Talaraich+Harvard-Oxford atlases and can easily be applied to images acquired in standard MRI protocols.

A hybrid version of the PSP atlas was evaluated, in which labelled subjects provided both T1w and QSM images, while unlabelled subjects only had T1w images. Despite being configured with a multimodal JLF framework, the hybrid version did not utilize the labelled QSM data in the absence of a corresponding unlabelled QSM image. Therefore, the label fusion for the unlabelled subjects relied on the T1w data only. The differences observed in the segmented ROIs between the T1w only and hybrid versions were largely not significant and likely due to the multimodal configuration used for the hybrid version. The hybrid version underperformed compared to both the T1w only and multimodal versions indicating that the benefits of multimodal fusion are only achieved when the additional imaging contrasts are available in the both the labelled and unlabelled data.

However, both the multimodal and T1w only versions of the PSP subcortical atlas greatly outperformed the Talaraich and Harvard-Oxford atlases and should be used in PSP neuroimaging analysis. Furthermore, the use of the PSP atlas is not limited to QSM and T1w data. Research in diffusion weighted MRI and tau PET studies have found significant results in subcortical regions (Dilcher et al., 2026; Li et al., 2024; Mitchell et al., 2019; Planetta et al., 2016). Currently these studies often use standard atlases or in-house templates based on manual segmentations despite the poor visualisation and delineation in these images. The PSP subcortical atlas could also be applied in these studies via the co-reregistration of the relevant images to the subjects labelled T1w image, making it relevant to research in all neuroimaging modalities and further highlighting the significant benefit and utility of the T1w only PSP atlas version.

The PSP subcortical atlas has several limitations. Firstly, the atlas was developed and validated using manual segmentations, however the segmentations were completed by a neuroimaging scientist and only reviewed by a senior neuroradiologist. Furthermore, as the segmentations were completed by only one person, inter-rater reliability metrics could not be calculated. This is a major limitation of the study, especially as the manual segmentations were used as the ‘gold standard’ to validate the atlases against. The manual segmentations were also completed on a homogenous dataset of PSP-RS subjects and has not been validated on other PSP phenotypes, limiting the generalisability of the atlas. The cohort also had a mean disease duration of 1.8 years since diagnosis and the atlas may not perform as accurately earlier or later disease stages. The PSP subcortical atlas was developed using multisite data, while making it generalisable across scanners and acquisition sequences this also introduces variability in the QSM images which may affect ROI visualisation for manual segmentations, impairing their reliability and reproducibility.

Future work will investigate if the PSP subcortical atlas can also be applied to PSP variants other than PSP-RS, and to other neurodegenerative diseases with similarly severe regional brain atrophy patterns and ventricular enlargement, such as frontotemporal dementia.

We created a PSP-specific atlas for subcortical brain regions. This atlas can be applied to PSP-RS subjects using both multimodal datasets and T1-weighted images alone. The PSP subcortical atlas outperforms currently available atlases and is a convenient alternative to manual segmentations. This atlas will greatly advance neuroimaging research in PSP-RS, with potential clinical applications, improving quality, replicability and efficiency of regional analyses and is currently being prepared for public availability as an Apptainer container.

## Data and code availability

The manual segmentations and code for the T1w only and multimodal versions of the PSP atlas are publicly available as an Apptainer container from https://github.com/iBrain-Lab/PSP_atlas.

## CRediT authorship contribution statement

**Cassandra Marotta:** Writing – review & editing, Writing – original draft, Methodology, Formal analysis, Data curation, Conceptualization. **Benjamin Sinclair:** Writing – review & editing, Supervision, Methodology, Conceptualization. **Meng Law:** Writing – review & editing, Visualization, Supervision, Methodology, Conceptualization. **Terence J. O'Brien:** Writing – review & editing, Supervision, Methodology, Funding acquisition, Conceptualization. **Lucy Vivash:** Writing – review & editing, Supervision, Methodology, Funding acquisition, Conceptualization.

## Funding

CM is supported by an Australian Government Research Training Program (RTP) stipend scholarship. BS has no disclosures to report. TJO reports funding from NHMRC Investigator Grants (APP1176426 and APP2034258) and Medical Research Future Fund (MRF2023250, MRF1170276, MRF2007605, MRF2024363 and MRF1200254) to support this work. TJO reports grant funding from NIH and DOD, consulting and fees and research grants (paid to his institute) from Kinoxis Therapeutics, Epidurex, UCB Pharma, Jazz Pharmaceuticals and Eisai for unrelated work. TJO reports fees for participation in advisory board from Kinoxis Therapeutics, Jazz Pharmaceuticals and UCB Pharma. TJO is on the Epilepsy Society of Australia committee but receives no honoraria. LV reports funding from NHMRC Medical Research Future Fund (MRF1200254) to support this work, and grant funding from Multiple Sclerosis Research Association, Department of Science Industry and Resources, Medical Research Future Fund (MRF1170276 and MRF2023250), Victorian Medical Research Accelerator Fund, Steminent (Taipei), Eisai (Japan) and Biogen (US), and provision of radiotracers from Life Molecular Imaging and Cyclotek for unrelated work. LV has received consultancy and scientific advisory board fees from Steminent for unrelated work, and has a current patent pending (PCT/AU2023/050497) for unrelated work.

## Declaration of competing interest

The authors declare that they have no known competing financial interests or personal relationships that could have appeared to influence the work reported in this paper.

## Data Availability

The manual segmentations and code for the T1w only and multimodal versions of the PSP atlas are publicly available as an Apptainer container from https://github.com/iBrain-Lab/PSP_atlas.
