# Supplementary material for: The PSP subcortical atlas - a new neuroimaging atlas for progressive supranuclear palsy Richardson's syndrome
Source: Neuroimage Clin. 2026 Sep 29;52:104075. doi: 10.1016/j.nicl.2026.104075 (PMC13659606; doi:10.1016/j.nicl.2026.104075)
Supplement: Supplementary file 1 [file mmc1.docx]

Supplementary table 1. MRI acquisition parameters

| **Site name** | Baker Institute, Prahran, Melbourne | Brain and Mind Centre, Sydney | Herston Imaging Research Facility (HIRF), Brisbane | South Australian Health and Medical Research Institute (SAHMRI), Adelaide | St Vincent’s Hospital Sydney | Macquarie University Hospital |
| --- | --- | --- | --- | --- | --- | --- |
| **Site number** | 01 | 03 | 04 | 05 | 06 | 07 |
| **Scanner** | 3T Siemens Prisma | 3T GE DISCOVERY MR750 | 3T Siemens Prisma | 3T Siemens Skyra | 3T GE Signa Architect | 3T Siemens MAGNETOM Vida |
| **Coil** | 64-channel head and neck coil | Nova 32-channel | 64-channel head and neck coil | 64-channel head and neck coil | 48-channel head coil | 64-channel head and neck coil |
| **3D T1-weighted magnetization-prepared rapid gradient echo (MPRAGE)** | | | | | | |
| **Voxel Size** | 0.8 mm isotropic | 0.8 mm isotropic | 0.8 mm isotropic | 0.8 mm isotropic | 0.8 mm isotropic | 0.8 mm isotropic |
| **TE** | 2.24 ms | 3.096 ms | 2.24 ms | 2.31 ms | 3.116 ms | 2.24 ms |
| **TR** | 2400 ms | 7.332 ms | 2400.0 ms | 2400.0 ms | 7.548 ms | 2400.0 ms |
| **TI** | 1060 ms | 450.0 ms | 1060 | 1060.0 | 450.0 | 1060 |
| **Flip angle** | 8° | 12° | 8° | 8° | 9° | 8° |
| **Slice thickness** | 0.8 mm | 0.8 mm | 0.8 | 0.8 | 0.8 | 0.8 |
| **Matrix size** | 320 x 300 | 320 x 320 | 320 x 300 | 320 x 300 | 320 x 320 | 320 x 300 |
| **FOV** | 240 x 256 mm | 256 x 256 mm | 240 x 256 mm | 240 x 256 mm | 240 x 256 mm | 240 x 256 mm |
| **Acceleration factor** | GRAPPA 2 | ASSET 2 | GRAPPA 2 | GRAPPA 2 | ASSET 2 | GRAPPA 2 |
| **Bandwidth** | 210 Hz/pixel | 260.438 Hz/pixel | 210 Hz/pixel | 210 Hz/pixel | 260.438 Hz/pixel | 211 Hz/pixel |
| **Acquisition plane** | Sagittal | Sagittal | Sagittal | Sagittal | Sagittal | Sagittal |
| **Number of slices** | 320 | 320 | 320 | 320 | 320 | 320 |
| **Susceptibility weighted imaging** | | | | | | |
| **Sequence** | 3D T2*-weighted multi-echo gradient echo with FLASH readout | Susceptibility Weighted Angiography (SWAN) Enhanced Fast Gradient Echo 3D | 3D T2*-weighted multi-echo gradient echo with FLASH readout | 3D T2*-weighted multi-echo gradient echo with FLASH readout | Susceptibility Weighted Angiography (SWAN) Enhanced Fast Gradient Echo 3D | 3D T2*-weighted multi-echo gradient echo with FLASH readout |
| **Voxel Size** | 0.83 mm isotropic | 0.83 mm isotropic | 0.83 mm isotropic | 0.83 mm isotropic | 0.8 mm isotropic | 0.83 mm isotropic |
| **TE** | 13.5 ms | 36.08 ms | 13.5 ms | 6.57 ms | 24.16 ms | 4.26 ms |
| **TR** | 24.0 ms | 46.9 ms | 24.0 ms | 24.0 ms | 35.5 ms | 24.0 ms |
| **Echo number** | N= 8. TE = 4.26, 6.57, 8.88, 11.19, 13.50, 15.81, 18.12, 20.43 ms | N= 7. TE = 7.0, 12.816, 18.632, 24.448, 30.264, 36.08, 41. 896 ms | N= 8. TE = 4.26, 6.57, 8.88, 11.19, 13.50, 15.81, 18.12, 20.43 ms | N= 8. TE = 4.26, 6.57, 8.88, 11.19, 13.50, 15.81, 18.12, 20.43 ms | N=5. TE= 6.0, 12.052, 18.104, 24.156, 30.208 ms | N= 8. TE = 4.26, 6.57, 8.88, 11.19, 13.50, 15.81, 18.12, 20.43 ms |
| **Flip angle** | 20° | 15° | 20° | 20° | 15° | 20.0° |
| **Slice thickness** | 0.83 | 0.8 | 0.83 | 0.83 | 0.8 | 0.83 |
| **Matrix size** | 288 x 216 | 320 x 320 | 288 x 216 | 288 x 205 | 320 x 320 | 288 x 218 |
| **Acceleration factor** | GRAPPA 2 | ASSET 2 | GRAPPA 2 | GRAPPA 2 | ASSET 2 | GRAPPA 2 |
| **FOV** | 288 x 270 mm | 256 x 256 mm | 288 x 270 mm | 213 x 239 mm | 256 x 256 mm | 227 x 240 mm |
| **Bandwidth** | 510 Hz/pixel | 223.188 Hz/pixel | 510 Hz/pixel | 510 Hz/pixel | 223.188 Hz/pixel | 511 Hz/pixel |
| **Acquisition plane** | Axial | Axial | Axial | Axial | Axial | Axial |
| **Number of slices** | 160 | 156 | 160 | 160 | 154 | 160 |

TE, echo time; TR, repetition time; TI, inversion time; FOV, field of view
